# Improved OTU-picking using long-read 16S rRNA gene amplicon sequencing and generic hierarchical clustering

**DOI:** 10.1186/s40168-015-0105-6

**Published:** 2015-10-05

**Authors:** Oscar Franzén, Jianzhong Hu, Xiuliang Bao, Steven H. Itzkowitz, Inga Peter, Ali Bashir

**Affiliations:** Department of Genetics and Genomic Sciences, Icahn School of Medicine at Mount Sinai, New York, NY USA; Division of Gastroenterology, Department of Medicine, Icahn School of Medicine at Mount Sinai, New York, NY USA

**Keywords:** Microbiome, 16S rRNA gene sequencing, OTU picking

## Abstract

**Background:**

High-throughput bacterial 16S rRNA gene sequencing followed by clustering of short sequences into operational taxonomic units (OTUs) is widely used for microbiome profiling. However, clustering of short 16S rRNA gene reads into biologically meaningful OTUs is challenging, in part because nucleotide variation along the 16S rRNA gene is only partially captured by short reads. The recent emergence of long-read platforms, such as single-molecule real-time (SMRT) sequencing from Pacific Biosciences, offers the potential for improved taxonomic and phylogenetic profiling. Here, we evaluate the performance of long- and short-read 16S rRNA gene sequencing using simulated and experimental data, followed by OTU inference using computational pipelines based on heuristic and complete-linkage hierarchical clustering.

**Results:**

In simulated data, long-read sequencing was shown to improve OTU quality and decrease variance. We then profiled 40 human gut microbiome samples using a combination of Illumina MiSeq and *Blautia*-specific SMRT sequencing, further supporting the notion that long reads can identify additional OTUs. We implemented a complete-linkage hierarchical clustering strategy using a flexible computational pipeline, tailored specifically for PacBio circular consensus sequencing (CCS) data that outperforms heuristic methods in most settings: https://github.com/oscar-franzen/oclust/.

**Conclusion:**

Our data demonstrate that long reads can improve OTU inference; however, the choice of clustering algorithm and associated clustering thresholds has significant impact on performance.

**Electronic supplementary material:**

The online version of this article (doi:10.1186/s40168-015-0105-6) contains supplementary material, which is available to authorized users.

## Background

Bacteria constitute the most abundant domain in the tree of life, and occur in virtually every habitat on earth. Body habitat-associated bacteria have received immense attention because of their relevance to human health and well-being [1, 2]. Until recently, these bacteria were largely studied with culture-dependent methods [3]. However, only a small fraction of all bacteria can be cultured in the laboratory. High-throughput DNA sequencing has bypassed the need to culture bacteria for assessing microbial diversity, enabling large-scale microbiome studies such as the Human Microbiome Project [4]. In a typical study, DNA is extracted from the whole community, and a target variable region of the small ribosomal subunit RNA gene (bacterial 16S rRNA gene or fungal 18S rRNA gene) is PCR-amplified with degenerate primers [5]. Millions of amplicons are sequenced and computationally analyzed to create profiles of bacterial richness, composition, and community structure. 16S rRNA gene is an ideal proxy for assessing bacterial diversity since it is universally conserved and relatively small (~1.5 kb) [6]. Importantly, 16S rRNA gene contains hypervariable regions that can provide species-specific signatures useful for identifying taxa. Most studies target only a few hundred base pairs of 16S rRNA gene, mainly due to limitations in sequencing read lengths. Such partial 16S rRNA gene sequencing can bias estimates of diversity, since nucleotide differences are not evenly distributed [7].

Operational taxonomic units (OTUs) are derived from clustering 16S rRNA gene (or fungal 18S) rDNA sequences and are used as approximations of microbial taxa [8]. OTU-based analysis is powerful in that it does not necessarily depend on a predefined database of 16S rRNA gene sequences and can theoretically resolve individual genomes. In contrast, taxonomy-dependent methods are limited by the reference database; currently, most reference databases are not characterized further than the genus level. In OTU analyses, 16S rRNA gene sequences are clustered at a certain level of sequence similarity, which approximates the taxonomic rank; e.g., >3 % dissimilarity is controversial but often used to define bacterial species [7–9]. Multiple methods have been proposed for binning 16S rRNA gene sequences into OTUs. Greedy heuristic methods (for example, *cd-hit* [10] and *usearch* [11]) reduce the search time and computational complexity at the cost of decreased accuracy. Such heuristics have a number of associated problems, including the tendency to create clusters that are more dissimilar than a specified threshold [12] and the inability to reconsider previous centroid selections, which generates spurious OTUs inflating taxonomic diversity. Hierarchical clustering (HC) algorithms are more accurate but slower since the computational complexity scales quadratically with the number of sequences [13], and multiple computational strategies have been developed to facilitate HCA-based OTU inference [14–16]. Schmidt et al. used ecological consistency as an external benchmark for cluster quality, and concluded that HC should be the default choice [17].

Nevertheless, the choice of binning strategy is an area of active research as it has been noted that different methods can deliver different partitions of the data. Ideally, the number of OTUs should reflect a certain taxonomic rank. A common artifact is overestimation of the number of OTUs as well as chimeric OTUs (consisting of sequences from two or more taxa). This is problematic since the number of OTUs is the most direct measurement of microbial diversity. While short 16S rRNA gene sequences (~100 to 400 bp) contain sufficient information to support high-quality OTU analysis in some datasets, longer sequencing reads have the potential to provide higher quality OTUs by covering a larger portion of, or the complete, 16S rRNA gene. Long-read sequencing is therefore a promising platform for characterizing samples with many phylogenetically close taxa, as commonly is the case with human microbiome samples. Furthermore, it has been shown that genetic distances computed on sub-regions of the 16S rRNA gene may significantly differ from those obtained using full-length sequences [18].

Single-molecule real-time sequencing (SMRT) from Pacific Biosciences (PacBio), can generate long reads (>10 kb) at relatively low cost per run (~$100/per chip as of 2015). As sequencing is performed on single molecules, the technology is insensitive to several types of context-specific biases arising from DNA amplification, such as GC-bias [19]. While the raw reads from PacBio have high error rates (>10 %), this can be alleviated using circular consensus sequencing (CCS) reads, in which the DNA polymerase reads the same DNA template multiple times [20]. The precise accuracy of the CCS reads depends on the quality and read length, where a higher number of fragment passes can lead to improved accuracy. With appropriate filtering, CCS reads are >99 % accurate [21], at the cost of limited throughput and sequencing length compared to raw reads. Notably, sequencing errors are independent of the location in the read (i.e., quality does not decrease further along the read, as is the case with Illumina and 454 sequencing). Marshall et al. demonstrated the use of PacBio CCS reads to characterize an electrosynthetic microbiota to the genus level [22], and a recent commentary describes the bioinformatic characteristics of PacBio CCS reads in more detail [21]. Another recent study performed amplicon sequencing of the 16S rRNA gene V1–V3 regions using PacBio CCS reads, and resolved OTUs to the genus level [23, 24]. Despite the application of CCS reads in several recent studies, the degree of improvement compared to short-read technologies is unclear, and there is currently a lack of bioinformatic guidelines for pre-processing and clustering of PacBio CCS reads.

In this study, we use simulated and experimental 16S rRNA gene data to evaluate the impact of read length and clustering algorithms on OTU analyses. Furthermore, we examine experimental 16S rRNA gene data focused on the genus *Blautia*, which are anaerobic members of the mammalian gut microbiota. *Blautia* spp. are believed to degrade complex polysaccharides to short fatty acids [25, 26] and have been linked to disease [27]. We undertake a mixed sequencing strategy on 40 human intestinal biopsy samples, utilizing short- and long-read sequencing of the 16S rRNA gene. We conclude that this mixed strategy is a cost-efficient way to profile novel taxa while taking advantage of the higher throughput of the Illumina MiSeq platform. Finally, we provide a flexible pipeline for sequence clustering, which parallelizes the most time-consuming steps by distributing jobs on a cluster.

## Results

### Simulated benchmarks show CCS reads improve OTU quality

**Fig. 1:**
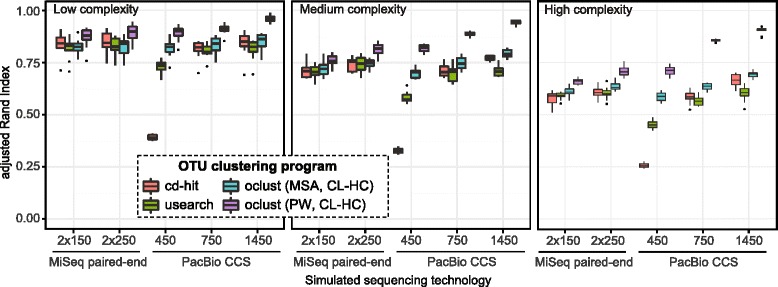
Boxplots of clustering accuracy of simulated sequencing on mock communities. Clustering accuracy was measured with the adjusted Rand index score (ARI; *y*-axis) on five simulated sequencing read lengths and four clustering programs. Values closer to zero indicate more dissimilar clustering compared to the ground truth and values closer to one indicate clustering in agreement with the ground truth. Simulated sequencing reads were generated on mock communities of low (left panel; 100 genomes/mock), medium (centered panel; 250 genomes/mock), and high (right panel; 500 genomes/mock) complexity. Each sequencing technology (*x*-axis; MiSeq: 2 × 150 and 2 × 250 bp paired-end reads, PacBio: 450, 750, and 1450 bp CCS reads) was simulated on 10 mock communities at each complexity level. Each box-and-whisker plot thus contains 10 observations. *Black dots* are outliers and the *centered horizontal line* inside the box corresponds to the median. *Red*, *green*, *blue*, and *violet* colors correspond to the programs *cd-hit*, *usearch*, *oclust* MSA (genetic distances computed from a multiple sequence alignment, and subsequent complete-linkage hierarchical clustering), and *oclust* PW (genetic distances computed from pairwise comparisons, and subsequent complete-linkage hierarchical clustering), respectively. Clustering was performed at 1 % increments from 1 to 6 % similarity. The best identity threshold per program and technology is shown

To assess whether long sequencing reads provide OTUs of higher quality compared with short sequencing reads, we simulated PacBio CCS reads (lengths 450, 750, and 1450 bp) and MiSeq paired-end reads (lengths 2 × 150 and 2 × 250 bp) on mock datasets of increasing complexity (Table 1). In silico amplicons were extracted to cover different regions of the 16S rRNA gene, and these were used as templates for generating synthetic reads. The V4 region is one of the most commonly targeted regions of the 16S rRNA gene, and therefore we included it in all amplicons, as it has also been noted to be one of the most informative. The V7 and V8 regions have been shown to be less informative. Amplicon lengths were distributed with the following medians: (V4) 414 bp, (V3–V4) 575 bp, (V1–V4) 781 bp, and (V1–V6) 1,466 bp. Slight variations in length occurred and were due to genome-specific differences in the 16S rRNA gene [28]. To compare clustering outcomes, we used the adjusted Rand index (ARI) [29], which is the corrected-for-chance version of the Rand index. ARI summarizes both precision (cluster purity) and recall (proportion of each genome partitioned into the same cluster). The gold standard is the reference genomes that are selected from the larger database. Each reference genome is selected from a filtered version of Greengenes; therefore, each mock community contains only unique genomes. Figure 1 and Additional file 1 show the distribution of ARI values for low (100 genomes/mock), medium (250 genomes/mock), and high (500 genomes/mock) complexity mock communities. Because the same identity threshold of two programs is not directly comparable, we report the best outcome of each program when clustering was performed at identity thresholds 1–6 % with 1 % increments. We further tested the model ARI~read length using linear regression followed by ANOVA. At each of the three complexity levels, there was a clear relationship between improved clustering outcome and longer reads (Fig. 1; Additional file 2). The *oclust* MSA algorithm was an exception, which did not indicate a linear relationship between ARI and read length at the low-complexity level (*p* = 0.61). The observed improvement in clustering outcome was dependent on the clustering method, where pairwise sequence comparisons followed by complete-linkage HC (*oclust* PW) outperformed DNACLUST (Additional file 3), *cd-hit*, *usearch*, and *oclust* MSA at all read lengths. The performance of *cd-hit*, *usearch*, and *oclust* MSA were similar at each of the three complexity levels. However, with PacBio CCS reads of length 450 bp, *cd-hit* leverages clustering which is much worse than the next best program (*usearch*). At the high complexity level, it becomes more difficult to distinguish genomes, as indicated by the lower ARI scores. At read lengths 750 and 1450 bp, *oclust* PW displays lower dispersion in the clustering outcome and the highest ARI scores (Fig. 1). Upon examination of OTUs formed by *oclust* PW, we found that the most common mistake was chimeric OTUs; singleton OTUs were less common. In the high complexity mock communities, the PacBio CCS reads provide the most significant improvement over short reads when clustering is performed with *oclust* PW.

**Table 1:** Details of the mock communities used for simulated sequencing

Complexity	No. mock communities	No. genomes per mock community	Total no. simulated sequencing reads
Low	10	100	100,000
Medium	10	250	250,000
High	10	500	500,000

While the number of OTUs does not directly measure accuracy, it is a more intuitive measurement than the ARI score and is widely used in the literature to assess α-diversity. We counted the number of OTUs formed for the high complexity mock communities at distance levels 1–6 % (Table 2). For the heuristic programs, we found that the number of predicted OTUs typically overestimate α-diversity. However, when clustering is performed with *oclust* PW, the number of OTUs decreased with increasing read length. Further examination of the composition of individual OTUs indicated that the heuristics generated more singletons or very small OTUs. PacBio CCS reads of 1450 bp length clustered with *oclust* PW at 1 % sequence similarity resulted in a median number of 529 OTUs, which was closest to the ground truth.

**Table 2:** Median numbers of inferred OTUs from the high complexity mock simulation

Program	cd-hit			usearch			oclust (MSA)			oclust (PW)		
Tech./seq. similarity^a^ (%)	1	2	3	1	2	3	1	2	3	1	2	3
MiSeq 2 × 150	2919	653	351	1758	477	327	2411	979	466	4287	1113	463
MiSeq 2 × 250	8085	2954	676	8413	3148	703	6544	2550	1041	8561	4011	1234
PacBio CCS 450	8555	4686	2449	8479	4870	2390	2053	940	546	1144	427	335
PacBio CCS 750	7910	1991	649	9356	5499	2319	2888	1152	594	757	399	349
PacBio CCS 1450	8488	1231	423	9874	6488	2327	2436	699	370	529	372	322

^a^The simulated technology (vertical); the similarity threshold used for clustering sequences into OTUs (horizontal)

### Comparing universal 16S rRNA gene primers with MiSeq and genus-specific primers with PacBio CCS

Forty intestinal tract samples were PCR-amplified using universal 16S rRNA gene primers. Pooled PCR products were sequenced with 2 × 250 reads on the Illumina MiSeq system, generating in total 6,422,050 read pairs (henceforth referred to as reads since the right and left reads were concatenated to a single sequence). After quality filtering and removal of human contamination, 594,019 reads passed chimera and quality filtering. The number of MiSeq reads per sample ranged between 3222 and 25,089 (mean number of reads per sample = 13,500; standard deviation of number of reads per sample [SD] = 4066). Sequences were taxonomically classified down to the genus level with *QIIME* [30], which identified the following number of taxa across all samples: 10 (phylum); 18 (class); 33 (order); 70 (family); and 145 (genus). As expected, bacteria of the phylum *Bacteroidetes* were the most represented (44.0 %; 261,507 reads), followed by *Firmicutes* (42.0 %; 249,741 reads) and *Fusobacteria* (3.4 %; 20,350 reads). At the genus level, *Bacteroides* were the most represented (38.2 %; 227,442 reads), followed by *Faecalibacterium* (11.7 %; 69,533 reads), *Blautia* (5.0 %; 29,989 reads), and *Fusobacterium* (3.4 %; 20,301 reads). The percentage *Blautia* per sample ranged between 0.6 and 15.9 % (mean = 5.4 %; SD = 3.2 %), consistent with the current knowledge of *Blautia* being a core taxon of the human gut microbiota despite its low abundance [31]. To assess reproducibility, we included four samples as replicates. Normalized abundance of taxa per sample was calculated at four taxonomic ranks (phylum, class, order, and genus), and Pearson’s *r* was used to evaluate the correlation (only taxa detected in both samples were considered). Mean correlation scores in the range 0.96 to 0.97 were observed from the four samples across all ranks: 0.97 (phylum); 0.97 (class); 0.97 (order); and 0.96 (genus) with SD = 1.9, 3.1, 3.5, and 2.8 %, respectively.

**Fig. 2:**
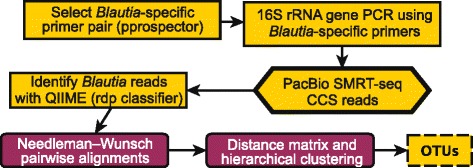
Flowchart of the OTU-picking pipeline. 16S rRNA gene primers specific for the *Blautia* genus were identified with *pprospector*, and the primer pair was used to generate 16S rRNA gene amplicons. Enriched PCR samples were targeted for SMRT-seq to get long CCS sequencing reads. CCS reads were taxonomically classified with *QIIME* pipeline, and reads classified to the genus *Blautia* were kept. Genetic distances were computed using pairwise alignments. Hierarchical clustering of the resulting dissimilarity matrix was performed at sequence similarities 1 to 6 % with 1 % increments. The final set of OTUs was filtered by requiring at least 10 or more supporting reads

**Fig. 3:**
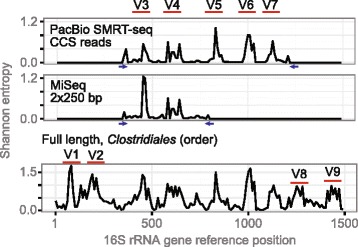
Shannon entropy along the 16S rRNA gene. Shannon entropy of PacBio CCS reads (*top*), MiSeq sequencing reads (*middle*), and full-length 16S rRNA sequences of the order *Clostridiales* (*bottom*). Shannon entropy was calculated from a multiple sequence alignment in 10 bp non-overlapping sliding windows. The *x*-axis shows the 16S rRNA gene reference position (i.e., the start position of the window), and the *y*-axis shows the average entropy signal for the window. *Horizontal red bars* indicate where the 16S rRNA gene hypervariable regions (V1 to V9) are located. *Blue arrows* indicate primer pairs. For reference, full-length sequences (*n* = 122,715) of taxa belonging to the order *Clostridiales* were extracted from *Greengenes* (*bottom*)

An overview of the experiment and analysis strategy is shown in Fig. 2. Based on 40 full-length 16S rRNA gene sequences of the genus *Blautia*, we designed a new degenerate primer pair to be used for genus-specific amplification (the primer pair is referred to as 404F/1263R). The primer pair produced an amplicon of ~800 bp and was confirmed to be specific by in silico matching toward full-length 16S rRNA gene sequences of other genera. 404F/1263R amplifies across five hypervariable regions (V3 to V7), whereas the universal primers only span across two hypervariable regions (V3 and V4). Subsequently, PCR amplification of the 44 samples was carried out using barcoded versions of 404F/1263R. The amplicons were pooled and then sequenced with PacBio SMRT-seq on eight SMRT-cells, yielding in total 417,538 PacBio CCS reads (at ≥3 passes). A negative relationship between the CCS read length and number of passes (Spearman’s rho = −0.25, *p* < 0.01) was observed. The median number of passes per CCS read was 15, and the bottom and top quartiles ranged between 3 to 9 and 24 to 153 passes, respectively. The CCS reads displayed a median length of 416 bp (median absolute deviation (MAD) = 336 bp), which indicated possible contamination or non-specific primer binding during the PCR amplification. A screen against the human genome identified 45 % (188,628/417,538) of the CCS reads as contaminants, and these were subsequently removed from further analysis. The remaining CCS reads were demultiplexed and then quality-, chimera-, and size-filtered (only keeping sequences ±100 bp of the expected amplicon size), resulting in 79,339 high-quality CCS reads with a median length of 826 bp (MAD = 7 bp). The number of passes for the high-quality CCS reads ranged from 5 to 39 (median = 16). Each sample contained from 206 to 3391 CCS reads (median = 1594 reads; mean = 1652 reads; SD = 725). The number of CCS reads classified to *Blautia* ranged from 16 to 1029 per sample (median = 255 reads; mean = 334 reads; SD = 234). In order to assess data quality, we evaluated the sequence diversity along the 16S rRNA gene. PacBio and MiSeq reads were aligned using a hand-curated 16S rRNA covariance model, and Shannon entropy was computed along the 16S rRNA gene (Fig. 3). Two and seven distinct peaks were seen for MiSeq and PacBio, respectively. Peaks colocalized with the known regions of hypervariable regions (V3 and V4 for MiSeq; V3 to V7 for the PacBio CCS), confirming the richer information content in CCS reads.

We next quantified the amount of taxonomic enrichment achieved, as measured by the mean fold change across samples and taxa using universal- and *Blautia*-specific primers. The level of enrichment ranged from two- to threefold and increased as the taxonomic ranks became narrower: 2.26× (phylum, *Firmicutes*); 2.30× (class, *Clostridia*); 2.30× (order, *Clostridiales*); 2.50× (family, *Lachnospiraceae*); and 3.10× (genus, *Blautia*). Relative abundances for each taxa level are shown in Additional file 4. While the fold change clearly indicated enrichment, we also observed enrichment of non-targeted taxa at the family and genus ranks. For example, only ~20 % of the PacBio CCS reads could be classified to the *Lachnospiraceae* family; the remaining enrichment was primarily observed in the following taxa (Additional file 5): *Ruminococcaceae*, *Erysipelotrichaceae*, *Clostridiaceae* (family); and *Faecalibacterium*, *Anaerostipes*, *Coprococcus*, *Roseburia*, *Ruminococcus*, *Subdoligranulum* (genera).

### OTU inference on experimental data

OTU profiles of the genus *Blautia* were investigated using *oclust* PW for the 40 human microbiome samples described above, consisting of 29,989 and 15,959 high-quality MiSeq and PacBio CCS reads, respectively. To improve our power to detect OTUs, we performed joint OTU inference, i.e., *Blautia* reads from all samples were pooled and treated as one sample during the clustering process. Table 3 summarizes the number of OTUs formed at similarity thresholds 1–6 % (1 % increments). Overall, MiSeq generated 1.86- to 3.06-fold more OTUs than PacBio CCS reads. Many inferred OTUs consisted only of one or a few sequences. While MiSeq generated overall more OTUs, PacBio CCS generated singleton OTUs more frequently than MiSeq; e.g., at 1 % sequence similarity, PacBio CCS reads generated 67.9 % singletons, whereas MiSeq generated 31.9 % singletons. Singleton OTUs can be interpreted as an indication of unsaturated sequencing, suggesting deeper sequencing could detect additional OTUs. This may indicate that CCS reads provide hints of yet to be discovered microdiversity. Singleton OTUs can also represent sequencing artifacts. We therefore applied a minimum of 10 reads per OTU, which dramatically reduced the number of OTUs, with more MiSeq OTUs observed except for the 1 % similarity threshold (237 OTUs formed from PacBio CCS reads vs. 106 OTUs formed from MiSeq reads). To confirm that the increase in MiSeq OTUs was not simply a result of deeper sequencing, we randomly selected 15,959 MiSeq reads and repeated the clustering (Table 3). With the exception of 1 and 2 % sequence similarity, MiSeq data still gave more OTUs than PacBio. Interestingly, PacBio CCS reads yielded 237 OTUs at 1 % sequence similarity, in contrast to 25 and 106 OTUs formed when MiSeq reads were sub-sampled and not sub-sampled, respectively. Notably, both technologies identified a flare-up in number of OTUs at 3 % sequence similarity, as commonly seen in human microbiota [9]. At a 6 % similarity threshold, we identified 88 OTUs within *Blautia* across 40 samples. To assess how this number compares to genera represented in a more comprehensive database, we counted unique genomes in the *Greengenes* database (processed as described in the “Methods” section) that have been classified at least to the genus level. We found that genera in this database contain a median number of 28 genomes per genus (median absolute deviation = 31). Given that this likely underrepresents true diversity, it suggests that 88 genomes within the *Blautia* genus are plausible.

**Table 3:** Number of inferred OTUs using oclust PW on PacBio CCS and MiSeq reads

	PacBio CCS reads		MiSeq 2 × 250 bp reads (full)		MiSeq 2 × 250 bp reads (sub-sampled)^a^	
Similarity threshold (%)	# OTUs (≥1 reads)^b^	# OTUs (≥10 reads)	# OTUs (≥1 reads)^b^	# OTUs (≥10 reads)	# OTUs (≥1 reads)^b^	# OTUs (≥10 reads)
1	4569 (3103)	237	11,362 (3634)	106	6636 (2412)	25
2	2282 (922)	171	6604 (725)	304	3938 (514)	103
3	1109 (237)	187	3397 (180)	1223	2056 (128)	616
4	532 (73)	186	1502 (62)	948	967 (50)	557
5	271 (22)	141	567 (25)	371	388 (23)	224
6	131 (9)	88	240 (12)	154	167 (11)	93

^a^Sequencing reads were sub-sampled to the same number of reads as PacBio CCS reads (*n* = 15,959)

^b^Number of singleton OTUs is specified within parenthesis

To further explore if sequencing errors are causing small OTUs, we examined the average quality score per PacBio CCS read of OTUs larger and smaller than 10 reads at 3 % sequence similarity. In total, 941 small OTUs (less than 10 reads per OTU) consisting of 2920 reads had an average quality score of 73.54 (SD = 4.51), whereas 168 large OTUs (*n* = 13,039 reads) had an average quality score of 74.39 (SD = 6.58), and the two distributions were significantly different (Kolmogorov-Smirnov (KS) test, *p* < 0.0001; *t* test, *p* < 0.0001). MiSeq reads displayed the same tendency (KS test, *p* = 0.047; *t* test, *p* = 0.027), although the number of reads distinguishing large (*n* = 27,635 reads; *n* = 1162 OTUs) and small (*n* = 2354 reads; *n* = 2235 OTUs) OTUs had to be decreased to five to get a statistically significant *p* value. Next, we performed the same comparison but explored if there was an indication of undetected chimeric reads. While we identified and removed chimeras prior to analyzing the data, undetected chimeras may remain. One way to find out is to compare the score distributions generated by uchime (each sequence is assigned a score indicating the chance of being a chimera). For OTUs based on PacBio CCS reads (using 10 reads to distinguish between large and small OTUs), the *uchime* score distributions were significantly different (KS test, *p* < 0.01; *t* test, *p* < 0.0001). The analogous analysis for MiSeq reads was also significant (KS test, *p* < 0.0001; *t* test, *p* < 0.0001). Hence, small OTUs are at least in part attributable to sequencing errors and possibly undetected chimeric reads. As a result, we used the threshold of at least 10 reads per OTU in downstream analysis to remove spurious OTUs. The cut-off resulted in substantially fewer OTUs (Table 3), though MiSeq OTUs were still more in numbers than PacBio CCS OTUs, except at the 1 % sequence similarity (MiSeq/PacBio CCS = 0.44). After filtering, the largest PacBio OTU at 3 % sequence similarity contained 2044 reads, and the median number of reads per OTU was 22.

Thresholding the number of passes for PacBio CCS reads, in principle, can be used to adjust the quality of input data. To investigate whether increasing the number of passes leads to improved OTU quality, we binned 15,959 PacBio CCS reads into four groups according to number of passes: (*i*) [3,9) passes, *n* = 1147 reads; (*ii*) [9,15) passes, *n* = 5999 reads; (*iii*) [15,24) passes, *n* = 6107 reads; and (*iv*) ≥24 passes, *n* = 2706 reads. Next, 1000 reads were randomly sampled from each group, and sequences were then clustered separately with *oclust* PW at 3 % sequence similarity. The number of OTUs formed from each group is listed in Additional file 6. There was no clear relationship between increased number of passes and delineated OTUs. In fact, the results suggest that increasing the number of passes does not lower the number of OTUs.

### Mixed-technology OTUs reveal further substructure from long reads

**Fig. 4:**
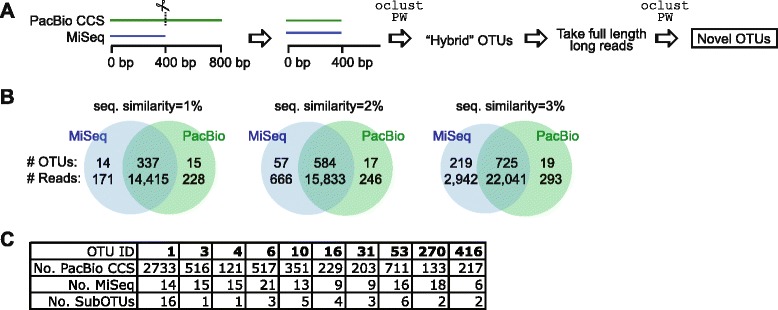
OTU substructure revealed by mixed-technology OTU-picking. **a** PacBio CCS reads were truncated to the same length as the short reads (covering the same region of the 16S rRNA gene). Short reads and truncated CCS reads were clustered into hybrid OTUs using the *oclust* PW pipeline (1–3 % sequence similarity). For shared OTUs, full-length CCS reads were clustered again. **b** Venn diagrams showing OTUs that were jointly identified by the two technologies. The intersection denotes hybrid OTUs, i.e., containing at least one sequencing read from each technology. Only OTUs with 10 or more sequences were counted. **c** Full-length PacBio CCS reads were extracted, and OTU-specific clustering was performed at 3 % sequence similarity. The number of sub-OTUs indicates how many additional OTUs were inferred

To investigate if long reads provide additional substructure, OTU inference was performed on PacBio CCS and MiSeq reads together, using sequences that were previously assigned to *Blautia.* First, PacBio CCS reads were trimmed to match the length of MiSeq reads (~400 bp), and MiSeq reads were sub-sampled to match the number of CCS reads (*n* = 15,959). Pairwise distances were computed for all 31,918 sequences, and OTUs were inferred at 1–3 % similarity with the *oclust* PW pipeline (Fig. 4a). In total, 8382 (similarity = 1 %; *n* = 3070 singletons), 4672 (similarity = 2 %; *n* = 698 singletons), and 2400 (similarity = 3 %; *n* = 200 singletons) OTUs were generated. The number of shared OTUs at different similarities is shown in Fig. 4b. Counting only OTUs with 10 or more reads, 92, 88, and 75 % OTUs were shared at similarities 1, 2, and 3 %, respectively; the lack of larger overlap may reflect inherent biases related to differential amplification by technology-specific primer pairs. Additionally, an unexpected number of MiSeq-specific OTUs were inferred at the 3 % similarity threshold (219 MiSeq vs. 19 PacBio). In terms of sequences, the numbers were higher: 97, 94, and 87 % sequences were incorporated into shared OTUs at similarities 1, 2, and 3 %, respectively. Thus, the number of OTUs that were MiSeq or PacBio-specific was relatively low. For the 10 OTUs with the highest number of trimmed PacBio CCS reads at similarity 3 %, we extracted and performed clustering at the same similarity on the full-length PacBio CCS reads. The results of the OTU-specific clustering are summarized in Fig. 4c. Eight of 10 OTUs revealed further substructure when clustering was performed on the full-length reads.

### OTU profiles and phylogenetic analyses of *Blautia* OTUs

**Fig. 5:**
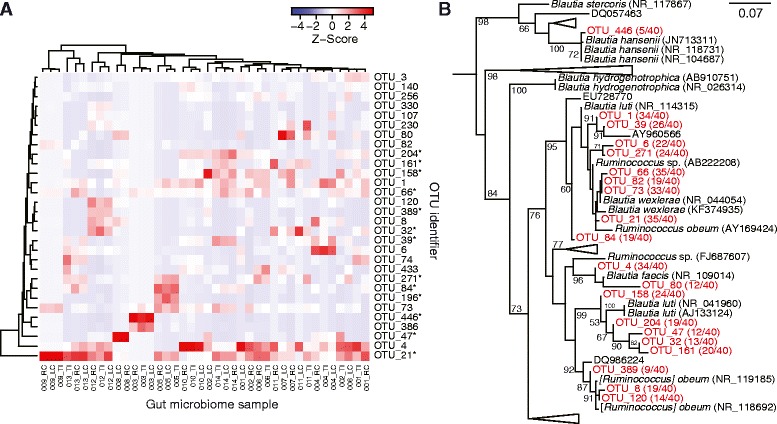
OTU profiles of the samples and phylogenetic analysis of *Blautia*. **a** Heat map of OTU profiles (*y*-axis) inferred from PacBio CCS reads at sequence similarity 3 % using *oclust* PW. The *x*-axis shows 40 human gut microbiome samples. Only the 30 most abundant OTUs are shown. For each sample, the number of reads per OTU was normalized by total number of reads from the sample. The normalized abundances were then further centered and scaled per sample to have mean zero and standard deviation one. The suffix of the sample label specifies the gut sub-location where the sample was taken from (TI = terminal ileum, RC = right colon, LC = left colon). The *asterisk* denoted OTUs matching sub-OTUs in Fig. 4c. **b** Maximum likelihood phylogenetic tree of representative sequences from *Blautia* OTUs. *Scale bar* on the top right corner indicates number of substitutions per site. Node support values >50 are shown. *Blautia* OTUs are indicated in red tip labels. *Black tip labels* represent sequences from GenBank, and the associated taxonomy information is shown if available. Taxonomic name/accession numbers are shown at the tips, and numbers in *parenthesis* refer to number of samples the OTU is present in. 16S rRNA sequences were aligned with *clustalw2* (40), and the tree was inferred using RAxML with the GTRGAMMA nucleotide substitution model and 1000 bootstrap replicates. The full tree is shown in Additional file 8

The relative abundance of the 30 most abundant OTUs, when applying *oclust* PW on full-length CCS reads, is displayed in Fig. 5a at 3 % sequence similarity, the typical threshold to delineate species [9]. When comparing with the mixed-technology OTUs presented in Fig. 4, we found that 13 out of the 30 most abundant OTUs had substructure unique to the longer PacBio CCS reads, not observed using the shorter MiSeq reads. Remarkably, none of the 1109 OTUs were universally present, suggesting an extensive diversity within the genus. The two largest OTUs consisted of 2044 (OTU_21) and 797 (OTU_4) CCS reads, respectively, explaining 17.8 % (2841/15,960) of the reads. OTU_21 and OTU_4 are present in all but six samples from the three subjects (008_LC, 008_RC, 003_RC, 003_TI, 003_LC, and 004_LC). Five of the samples from the two subjects missing OTU_21 and OTU_4 (008_LC, 008_RC, 003_RC, 003_TI, and 003_LC) instead showed signals of OTU_446 and OTU_386. Since sample biopsies were taken from three different intestinal locations, we examined if the location was correlated with the presence of one or more OTUs. However, a multidimensional scaling plot of the abundance matrix did not indicate clustering according to sample site (Additional file 7). Moreover, there was little difference in OTU abundances between the three sites (Kruskal-Wallis test; all *p* values >0.29). These data suggest the identified *Blautia* taxa do not preferentially colonize one or more of the three locations, and there is no evidence for an abundance gradient in the intestine. Next, we determined phylogenetic affinities of the inferred OTUs by assuming that OTUs are monophyletic. The analysis was limited to “large” OTUs (*n* = 20), here defined as OTUs with ≥150 CCS reads. The prevalence of these OTUs ranged 12.5–87.5 % (SD = 21.9 %), of which OTU_4 and OTU_21 were present in 87.5 and 85.0 % of the subjects, respectively. One representative sequence was selected from each OTU by computing the mean genetic distance per sequence to all other sequences in the OTU, and selecting the sequence with the minimum mean distance. As a result, 20 representative CCS reads were extracted and queried through GenBank (non-redundant nucleotide collection, excluding uncultured and environmental sequences). These sequences were placed relative to a reference phylogeny including 194 near full-length 16S rRNA sequences, of which 92 represented non-redundant taxa. A maximum likelihood phylogenetic tree was created and rooted using *Caldicoprobacter guelmensis* as an outgroup species (Fig. 5b; the complete tree is shown in Additional file 8). We observed the following: OTU_446 was placed within a clade of sequences annotated as *Blautia hansenii* (bootstrap support (bs) = 100; previously named *Ruminococcus hansenii* (18)); OTU_1 and OTU_39 cluster relatively close to *Blautia wexlerae* (bs = 91; previously *Ruminococcus luti* (18)); OTU_66, OTU_82, OTU_73, and OTU_21 were close to *Blautia wexlerae* and *Ruminococcus obeum* (bs = 33–78); and OTU_4 is close to *Blautia faecis* (bs = 96). While the OTUs cluster close to known reference sequences, each OTU of *Blautia* may include more than one species, as only one representative sequence was used for clustering.

### qPCR validation

*Blautia* OTUs identified from long reads using *oclust* were further validated by qPCR using OTU-specific primers. Based on the total amount of reads (Fig. 4) of each identified OTU, the dominant OTU_21 was selected to be validated by qPCR. We found the long-read sequencing and qPCR results to be correlated (Pearson’s *r* = 0.33, *p* value = 0.019).

## Discussion

In this study, we take advantage of previous algorithmic developments for microbial community analysis, and demonstrate the effectiveness of PacBio CCS reads for OTU inference. Our study achieves improved OTU quality by pairwise alignments of CCS reads followed by complete-linkage HC. While PacBio CCS has lower sequencing throughput than the MiSeq platform (the former generated between 10,000 and 20,000 reads in our study, though current chips can exceed 50,000 reads), its advantage comes from the long-read length. By focusing on the single genus *Blautia* using custom 16S rRNA gene primers, we were able to take advantage of generic HC, providing more exact binning of sequences into OTUs. In contrast to heuristic methods, HC avoids the input-order dependency problem, i.e., different input order of sequences can yield different clustering outcomes. Furthermore, HC is algorithmically simple as it only requires a pairwise distance matrix, and the agglomerative nature makes it trivial to test multiple cut-offs once complete clustering has finished [16, 32, 33]. Nevertheless, heuristic methods (such as *cd-hit* and *usearch*) represent attractive alternatives in cases where HC is computationally infeasible (e.g., when sequence numbers are in the order of millions). In our study, we evaluated HC when genetic distances were computed from pairwise alignments and multiple sequence alignments (MSA). Our data indicate that pairwise alignments followed by complete-linkage HC outperform all other tested methods. On the contrary, MSA-based computation of genetic distances followed by HC did not perform better than the heuristic methods. While we have compared two popular heuristics, this study is not an exhaustive comparison of OTU-picking algorithms. For example, we note that the speed of the pairwise alignments can be greatly improved by implementing a *kmer*-based distance filter similar to ESPRIT [15] and SWARM [18], in order to avoid unnecessary comparisons. Moreover, Sun et al. provides a convenient solution for performing complete-linkage HC without loading the full distance matrix into the random access memory [15].

Our results show a profound increase in OTU quality as the read length increased. This effect was captured by both heuristic methods and HC, although pairwise alignments combined with HC produces the most pronounced improvement. Importantly, the improvement in OTU quality was related to the complexity of the mock dataset, the choice of OTU-picking algorithm, and the choice of global clustering threshold. As with other OTU-based methods, the challenge is to find a distance threshold that maps to a certain taxonomic level. We set our mock samples with a fixed degree of abundance per taxon. In reality, taxon abundance is variable and evolutionary rates are not constant across lineages, suggesting a single clustering threshold may be difficult to establish. We therefore propose a combined sequencing strategy where limited taxa are investigated, as it becomes more likely that the evolutionary rates are similar.

The impact of read length was also apparent when analyzing the *Blautia* experimental data. A number of OTUs were able to be further refined via the addition of long-read data. These sub-OTUs were sometimes some of the most frequently observed species within patients, and played an important role in correctly clustering individuals (as shown with clusters 204, 161, 158, 66, 39, and 21 for individual 14 in Fig. 5a). Overall, we found a remarkable diversity within *Blautia*, of which our results suggest much diversity is still to be discovered. While *Blautia* has been previously explored using oligotyping [26], those results are not directly comparable due to differences in OTU-picking strategy. Most likely, many of our identified OTUs reflect strains or subspecies diversity, i.e., at the 3 % sequence similarity, we identified 1109 OTUs, which may not be reflective of distinct species. While our data suggests that the human gut contains more than one species of *Blautia*, and that two OTUs with similarity to *Blautia wexlerae* and *Blautia faecis* are the most abundant, it is impossible to select a single threshold for genetic distance for grouping and translating it to a position in Linnaean taxonomy. In fact, recent studies have begun to shed light on phylogenetic inconsistencies in the OTU concept—OTUs may contain sequences from multiple taxa and represent different strains of the same species [32, 33]. Though increased read lengths and appropriate algorithms for OTU picking can dramatically improve OTU demarcation, we may still observe an overestimation of OTUs independent of clustering method and read length. However, as shown in *Blautia*, whether or not these sequences represent distinct species, there is clearly a tremendous amount of additional phylogenetic information that is missed without complete (or near complete) examination of the 16S rRNA gene interval. As throughput and accuracy of PacBio sequencing continue to improve, we anticipate full-length 16S rRNA sequencing to become an important tool for full microbial community profiling.

## Conclusions

Together, our simulated and experimental data demonstrates that long reads can improve OTU inference; however, the choice of clustering algorithm and associated clustering thresholds has significant impact on performance.

## Methods

### Human specimens

Mucosal biopsies were obtained from patients with inflammatory bowel disease already undergoing colonoscopy for clinical purposes under a protocol approved by the Icahn School of Medicine Institutional Review Board. All patients signed informed consent. Specimens were taken from the terminal ileum (TI), right colon (RC), and left colon (LC) from each individual when possible.

### Clustering of 16S rRNA gene sequences into OTUs

#### Hierarchical clustering (oclust)

Given our comparatively low CCS read depth, it was computationally feasible to implement generic HC, completely avoiding heuristic methods. The first step in the pipeline oriented sequences to the same strand by querying sequences with BLAST (v.2.2.26; settings: -v 1 -b 1 -e 1e-10 -FF) toward the first 10,000 entries of the *Greengenes* database (v.13.5.99), and reverse-complemented if necessary. Subsequently, genetic distances were computed by all-versus-all pairwise sequence alignments using the Needleman-Wunsch (NW) algorithm [34] implemented in the emboss program *needle*. Single base insertions and deletions (indels) are the most common errors in PacBio CCS reads [35], and such errors may artificially inflate OTU estimates if included when computing genetic distances. Alignments were pre-processed by removing terminal alignment gaps and internal single base indels. The genetic distance was then computed as the pairwise identity, i.e., the number of mismatches divided by the number of examined columns. Alignment gaps were regarded as indels and each indel was counted once. Genetic distances were converted to dissimilarity matrices, which were used for HC. In addition to all-versus-all pairwise alignments, we investigated the utility of secondary structure-aware MSA for computing genetic distances. Such methods have been implemented using covariance models and Hidden Markov Model-accelerated techniques in the program *Infernal* [36]. The advantages of this strategy include reduced computational footprint (sequences can be aligned within reasonable time and memory on a desktop computer) as well as the incorporation of secondary structure information in the alignment. We used *Infernal* v.1.1rc4 and a hand-curated bacterial 16S rRNA MSA (http://rdp.cme.msu.edu/download/RDPinfernalTraindata.zip). The latter was formatted with the command “cmbuild --ere 1.4,” and sequences were aligned with cmalign to this model (default settings). Distances were computed with the dist.alignment function of the *seqinr* R package, and subsequently squared as this function returns the square root of the genetic distance.

Clustering was performed in *R* using the function “hclust” (complete-linkage), and the final clustering was obtained by running the function “cutree” on the object returned by “hclust.” The height at which the tree is cut defines the clustering threshold, and it is comparable with percent identity in heuristic OTU-clustering methods. The optimal HC-linkage function is a debated topic [12], and previous studies have suggested average-linkage to be more accurate than complete-linkage clustering [12, 37-39]. Single-linkage clustering is rarely used in OTU-based methods due to its chaining effects [12, 40]. We compared average-, complete-, and single-linkage clustering. Based on the comparison results (Additional file 1), we used the complete-linkage as the default choice for *oclust*. Complete-linkage clustering has further been shown to provide the most ecologically consistent partition of 16S data [17].

#### Heuristic methods

Sequences were clustered into OTUs using *cd-hit* v.4.6.1 [10], *usearch* v.7.0.1001 [41], and DNACLUST v.3 [42]. The programs were invoked using the following command line parameters: “cd-hit-est -mask NX -n 8 -l 11 -p 1 -d 0 -g 1 -r 1” (cd-hit); “-cluster_fast” (usearch); and “-l -k 3” (DNACLUST).

### Simulated sequencing

#### Mock communities

Reference 16S rRNA sequences (genomes) were used to simulate reads, and these were selected from *Greengenes* (v.13.5.99) [43]. Initially, the database was filtered to contain only unique sequences >1400 bp in length, which reduced the number of genomes from 1,075,170 to 338,271. We further limited the selection space only to genomes classified to the phylum *Bacteroidetes*, which is one of four dominant phyla present in most mammalian microbiotas. To avoid bias caused by overrepresentation of certain taxa, we randomly down-sampled genera with more than 1000 genomes to 100. We then created mock communities at three levels of increasing complexity by randomly selecting genomes (Table 1): low (*n* = 100 genomes), medium (*n* = 250 genomes), and high complexity (500 genomes). We created 10 mock communities at each complexity level in order to obtain an estimate of the statistical dispersion. Accession numbers for genomes included in the mock communities are listed in Additional file 9.

#### Synthetic sequencing reads

*In silico* amplicons were extracted from mock genomes by first creating one MSA containing all reference sequences. Sequences were aligned with *Infernal* as above, and subsequences (amplicons) were extracted from the following 16S rRNA alignment positions: pos. 389–801 (covering V4; MiSeq 2 × 150); pos. 227–801 (covering V3–V4; MiSeq 2 × 250 and PacBio 450); pos. 4–801 (covering V1–V4; PacBio 750); and pos. 4–1506 (covering V1–V6; PacBio 1450). We used *ART* v.2.3.7 [44] and *pbsim* v.1.0.2 [45] to simulate synthetic sequencing reads for MiSeq and PacBio, respectively. The ART-provided quality profile was used to generate MiSeq 2 × 150 bp (settings: -amp -l 150 -f 100) and 2 × 250 bp (-l 250 -f 100) synthetic-paired reads. The second sequence in the pair was reverse-complemented and concatenated with the first sequence but separated with a 10 nucleotide “N” spacer. For *pbsim*, we used the bundled CCS quality model and the following settings “--accuracy-mean 0.99 --accuracy-sd 0.01 --difference-ratio 6:21:73 --data-type CLR --model_qc model_qc_ccs --depth 50.” We subsequently down-sampled each genome to have exactly 20 synthetic reads. To ensure that the order in which sequences occur does not influence the clustering outcome, we randomly shuffled sequences prior to clustering them.

#### Evaluating clustering quality

Clustering quality was measured using total number of inferred OTUs and the ARI [29] as calculated in the *mclust* R package. ARI penalizes chimeric and duplicate OTUs and allows comparison of clustering results between thresholds and programs. An ARI score of 1 indicates complete agreement with ground truth.

### Experimental data

#### MiSeq

In total, 40 biopsies from LC, RC, and TI were collected and snap frozen immediately after collection. Four biopsies from two subjects were processed twice using the same protocols described below to evaluate the overall performance of the MiSeq system for the 16S rRNA gene sequencing. Total DNA was extracted from the fresh frozen tissue biopsies using the UltraClean Tissue & Cells DNA Isolation Kit (MO-BIO, CA). The 16S rRNA gene was then amplified by PCR with 16S rRNA gene 8-base double-barcoded 347F/803R primer pairs. The integrity of the amplicons was verified by agarose gel electrophoresis. The resulting ~460 bp-sized amplicons were pooled and then sequenced with the MiSeq 2 × 250 paired-end system. The sequence data were trimmed and quality filtered in the following way: the last 42 bp of the second read were discarded, and the resulting sequence was reverse-complemented and merged with the first read separated by a 42 bp N spacer. Quality filtering was subsequently performed and only sequences with quality score ≥30 over at least 97 % of the length (excluding the spacer sequence) were kept. Chimeras were detected as described below for PacBio CCS reads. Barcodes were identified and removed using the program *scan_for_matches* (http://blog.theseed.org/servers/2010/07/scan-for-matches.html, R. Overbeek) with zero mismatches allowed in the barcode sequence.

#### PacBio

*Blautia*- and OTU-specific primers were selected using *pprospector* v.1.0.0 [46]. *Blautia* reference sequences (*n* = 40) were extracted from *Greengenes* (v.12_10), and aligned with *Infernal* as above. Accession numbers for the *Blautia* sequences are listed in the “Accession numbers” section below. The 12-base-barcoded 404F/1263R primer pairs were designed based on 16S rRNA reference sequence of *Blautia* genus, and the ~860 bp-sized PCR amplicons were pooled for sequencing on the PacBio RS II. Sequencing data from PacBio was processed using *smrtanalysis* v.2.1.1 (https://github.com/PacificBiosciences/SMRT-Analysis/). CCS reads were then checked for human contamination by querying sequences with *megablast* v.2.2.26 (low-complexity filter turned off) [47] toward the human reference genome (hg19). Reads deemed to be contaminated had E-values <1e−05, and these were subsequently discarded. Quality filtering of the PacBio CCS reads was performed using the associated phred-like quality values, which were extracted from the fastq files and converted from ASCII to integers. The threshold for quality filtering was set to >Q30 across 90 % of the sequence. Chimeras were identified and removed with *uchime* v.4.2.40 [48] in reference mode with the option “--minh 1.0.” The ChimeraSlayer reference database was used. The threshold for classifying a sequence as chimeric was determined by running the program on simulated PacBio data and selecting the threshold for which there are no false positives.

### Entropy analysis

Sequencing reads were aligned with *Infernal* (as above). Alignment columns representing insertions in the target sequence relative to the reference were removed. Shannon Entropy (*H*) was calculated for every sequence position as follows:

$$ {H}_i=-{\displaystyle \sum}\left({f}_{ai}\times { \log}_2{f}_{ai}\right) $$

where *f* is the relative frequency of base *a = {A*, *T*, *G*, *C}* at position *i* of a sequence of length *n*. The average *H* was then calculated in 10 bp non-overlapping windows along the 16S rRNA gene.

### Taxonomic classification

Taxonomic classification of sequences was performed with the “assign_taxonomy.py” command in *QIIME* version 1.4.0 [30] using the *rdp classifier* v.2.6 [49] as the underlying method. Taxa with confidence score >0.8 were considered significant. The family rank of *Blautia* was changed from “Incertae Sedis XIV” to *Lachnospiraceae* to reflect the most recent taxonomic classification [50].

### Phylogenetic analysis

*Blautia* sequences were identified in NCBI GenBank using BLAST, and aligned with *Infernal* [36], *mafft* v7.029b [51], *kalign2* [52], and *clustalw2* [53], respectively. We then inferred one phylogenetic tree for each multiple alignment using *RaXML* v7.9.3 [54] with the GTRGAMMA model and 1000 bootstrap replicates. We subsequently computed the median of the node support values for each tree and selected the tree with the highest median bootstrap support, which was the tree based on the multiple alignment from *clustalw2*.

### Scripts availability

The *oclust* pipeline is implemented in Perl and R, and automatically performs pre-processing and clustering of sequences using distance matrices computed from pairwise alignments or MSAs. *oclust* requires a Linux x86-64 system, and can be downloaded from https://github.com/oscar-franzen/oclust/.

### Real-time quantitative PCR

One-step real-time qPCR was performed on a LightCycler 480 PCR system (Roche, CA) using the SYBR Advantage qPCR Premix kit (Clontech, CA) with modified cycling conditions. Following incubation at 95 °C for 1 min, required for the hot start activation of DNA polymerase, amplification was carried out for 40 cycles using 95 °C for 10 s, 55 °C for 10 s, and 72 °C for 10 s. Bacterial 16S rRNA was directly amplified using universal 16S rRNA gene primers and *Blautia*-specific primers. The abundance of the rare OTUs was below the PCR detection limit and therefore excluded from the validation. At the end of each reaction, crossing point (Cp) corresponded to the first peak of a second derivative curve, and melting curves were acquired and analyzed to validate the products. The abundance of OTU_21 in each sample was determined by normalizing the Cp value to the Cp value obtained using the universal 16S rRNA gene primers. The association between paired OTU abundances from sequencing and qPCR was tested using Spearman’s rho.

### Primers

The following degenerate primer pairs were used for simulated sequencing (designation, variable regions covered, approximate amplicon size): GTGCCAGCMGCCGCGGTAA, GGACTACHVGGGTWTCTAAT (A, V4, 300 bp); GGAGGCAGCAGTRRGGAAT, GGACTACHVGGGTWTCTAAT (B, V3–V4, 500 bp); AGAGTTTGATYMTGGCTCAG, CTACCRGGGTATCTAATCC (C, V1–V4, 750 bp); and AGAGTTTGATYMTGGCTCAG, GGTTACCTTGTTACGACTT (D, V1–V6, 1450 bp). For the real 16S rRNA gene sequencing and the real-time PCR, all the PCR primers were synthesized by *IDT* (Integrated DNA technology, IA).

### Accession numbers

The following sequences were downloaded and used for primer design (note that some of these sequences have a different taxonomic annotation in NCBI GenBank/rdp classifier; we, however, used the taxonomic classification from *Greengenes*): AB185576.1, AJ270469.2, AJ413954.1, AJ508452.1, AY169422.1, AY442822.1, AY854272.2, DQ793887.1, DQ794453.1, DQ794525.1, DQ795214.1, DQ797229.1, DQ797752.1, DQ797854.1, DQ798179.1, DQ798613.1, DQ799717.1, DQ799837.1, DQ800053.1, DQ800661.1, DQ801118.1, DQ802725.1, DQ805401.1, DQ806241.1, DQ806770.1, DQ806799.1, DQ806910.1, DQ807741.1, DQ807831.1, DQ808081.1, DQ809215.1, DQ809319.1, DQ809896.1, DQ810148.1, DQ823680.1, DQ824124.1, DQ824213.1, DQ905770.2, X85101.1, and Y10584.1

## Additional files

Additional file 1:
**Comparison of hierarchical clustering algorithms.** Average- (AL), complete- (CL), and single (SL)-linkage hierarchical clustering were evaluated on low, medium, and high complexity mock communities using the adjusted Rand index (ARI; *y*-axis). The clustering outcome was then evaluated at 11 distance thresholds (0.5–10 %). Red, green, and blue colors correspond to AL, CL, and SL, respectively. Additional file 2:
**Effects of read lengths on clustering of simulated data.** Linear regression tests were performed on ARI versus read lengths, and significance testing was performed using analysis of variance (ANOVA). *p* values for each program and mock community are listed in column three. Additional file 3:
**Clustering accuracy of simulated sequencing on mock communities using DNACLUST.** Clustering accuracy was measured with the adjusted Rand index score (ARI; *y*-axis) on five simulated sequencing read lengths by DNACLUST. The performance of DNACLUST is similar to the two other heuristics at all complexity levels. Additional file 4:
**Taxonomic enrichment achieved within** ***Blautia.*** The *y*-axis shows the mean percentage reads classified to a specific taxonomic rank per sample. Error bars correspond to the standard deviation across samples. The *x*-axis shows the taxonomic rank along the *Blautia* lineage. Taxonomic classification was performed with QIIME (scores >0.8 were considered correct). Black and gray colors refer to universal primers and *Blautia*-specific primers, respectively. Additional file 5:
**Non-specific taxonomic enrichment.** Bar plots showing enriched taxa for sequencing using universal 16S rRNA gene primers (MiSeq) as compared with *Blautia* (genus)-specific primers (PacBio CCS). The *y*-axis refers to percentage of sequences in all samples. The *x*-axis refers to the specific taxa. The left plot shows taxa at the family level, and the right plot shows taxa at the genus level. Only taxa with abundance >0 using universal- and *Blautia*-specific primers are shown. Data (*y*-axis) were square root transformed prior to plotting. Additional file 6:
**Number of OTUs formed from binning of PacBio CCS reads by number of passes.** PacBio CCS reads were binned into four groups by the number of CCS passes. The bins correspond to the following number of passes: bin 1 [3,9); bin 2 [9,15); bin 3 [15,24) reads; and bin 4 ≥ 24. Additional file 7:
**Multidimensional scaling plot of intestinal sample locations versus OTU profiles.** *Blautia* OTU profiles were computed with *oclust* PW (sequence similarity = 3 %), and multidimensional scaling was performed in *R* using the cmdscale command. Colors refer to the intestinal location (red = LC, green = RC, blue = TI). Sample identifiers are plotted below each point. Additional file 8:
**Maximum likelihood phylogenetic tree of representative 16S rRNA gene sequences of** ***Blautia*** **OTUs.** One representative sequence from each OTU was selected using the minimum genetic distance. Closely related full-length 16S rRNA sequences were identified from NCBI GenBank. A multiple sequence alignment was created with clustalw2, and the phylogeny was inferred with RAxML with the GTRGAMMA nucleotide substitution model. Node labels indicate bootstrap support values. If the reference sequence from GenBank had meaningful taxonomic information (e.g., binomial species name), it is shown on tips; otherwise, only the accession number is shown. Square brackets around a genus name indicate a candidate genus. The top left scale bar indicates number of substitutions per site. The tree was rooted using *Caldicoprobacter guelmensis* (NR_109614). *Blautia* OTUs are indicated in red and the identifier of the representative PacBio CCS read is shown in parenthesis. Additional file 9:
**Genomes included in the sequencing benchmark.** Table of accession numbers of 16S rRNA gene sequences used for mock communities (each row corresponds to one 16S rRNA gene sequence). Columns: (*i*) complexity level of the mock community; (*ii*) identifier of the mock community; (*iii*) *Greengenes* identifier of the record; (*iv*) the NCBI GenBank accession number for the record; (*v*) and the taxonomic classification of the genome.
