# Erratum to: Improved OTU-picking using long-read 16S rRNA gene amplicon sequencing and generic hierarchical clustering

**DOI:** 10.1186/s40168-015-0123-4

**Published:** 2015-10-28

**Authors:** Oscar Franzén, Jianzhong Hu, Xiuliang Bao, Steven H. Itzkowitz, Inga Peter, Ali Bashir

**Affiliations:** Department of Genetics and Genomic Sciences, Icahn School of Medicine at Mount Sinai, New York, NY USA; Division of Gastroenterology, Department of Medicine, Icahn School of Medicine at Mount Sinai, New York, NY USA

## Erratum

After publication of this article [1], the authors noticed one of the funding sources had been omitted from the ‘Acknowledgements’ section. Helmsley Foundation also helped to fund this work.

Please find the correct version of the ‘Acknowledgments’ section below:
