# Supplementary figures and images for: Improved OTU-picking using long-read 16S rRNA gene amplicon sequencing and generic hierarchical clustering
Source: Microbiome. 2015 Oct 5;3:43. doi: 10.1186/s40168-015-0105-6 (PMC4593230; doi:10.1186/s40168-015-0105-6)

# oclust PW - LC

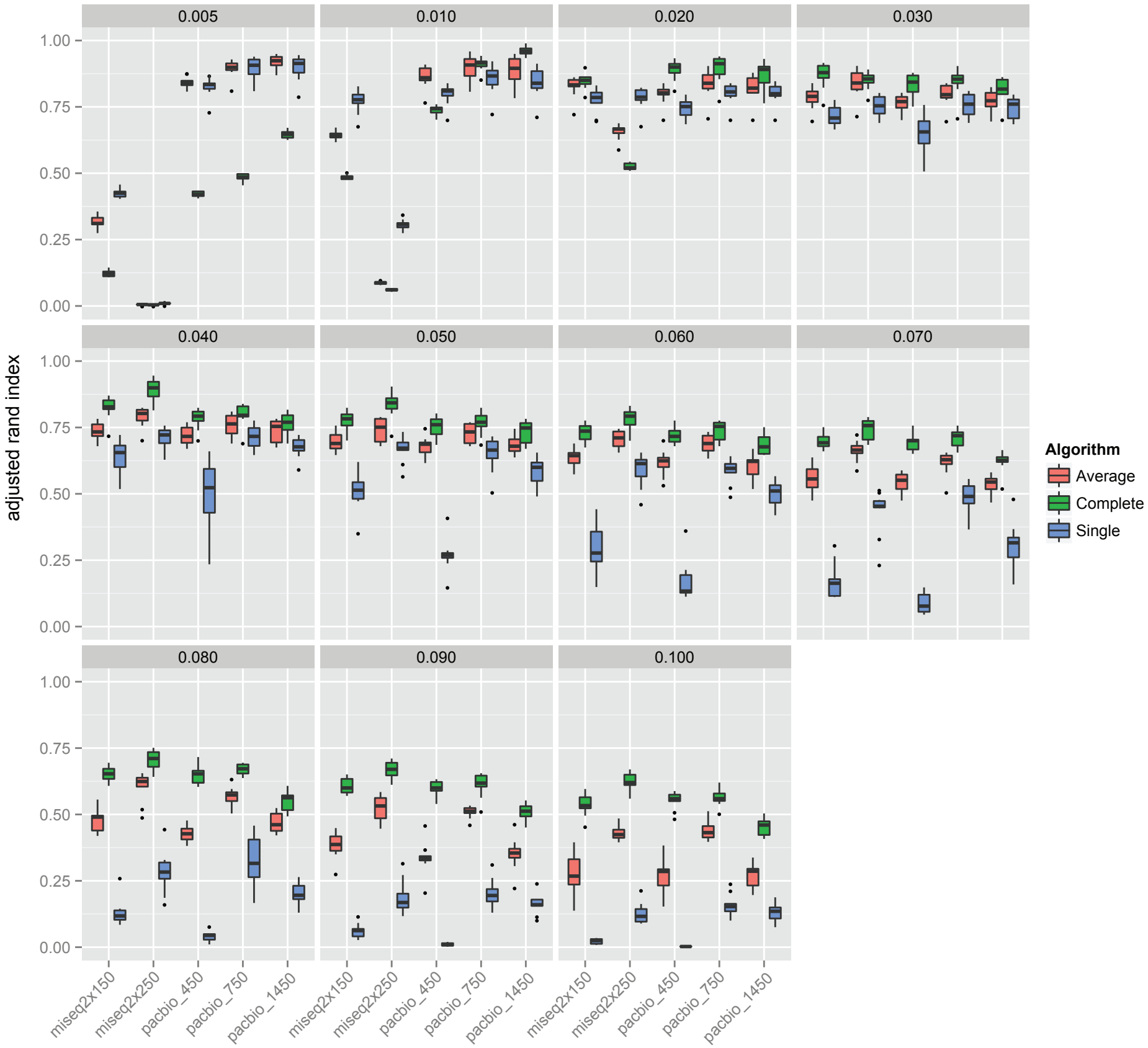

oclust PW - MC

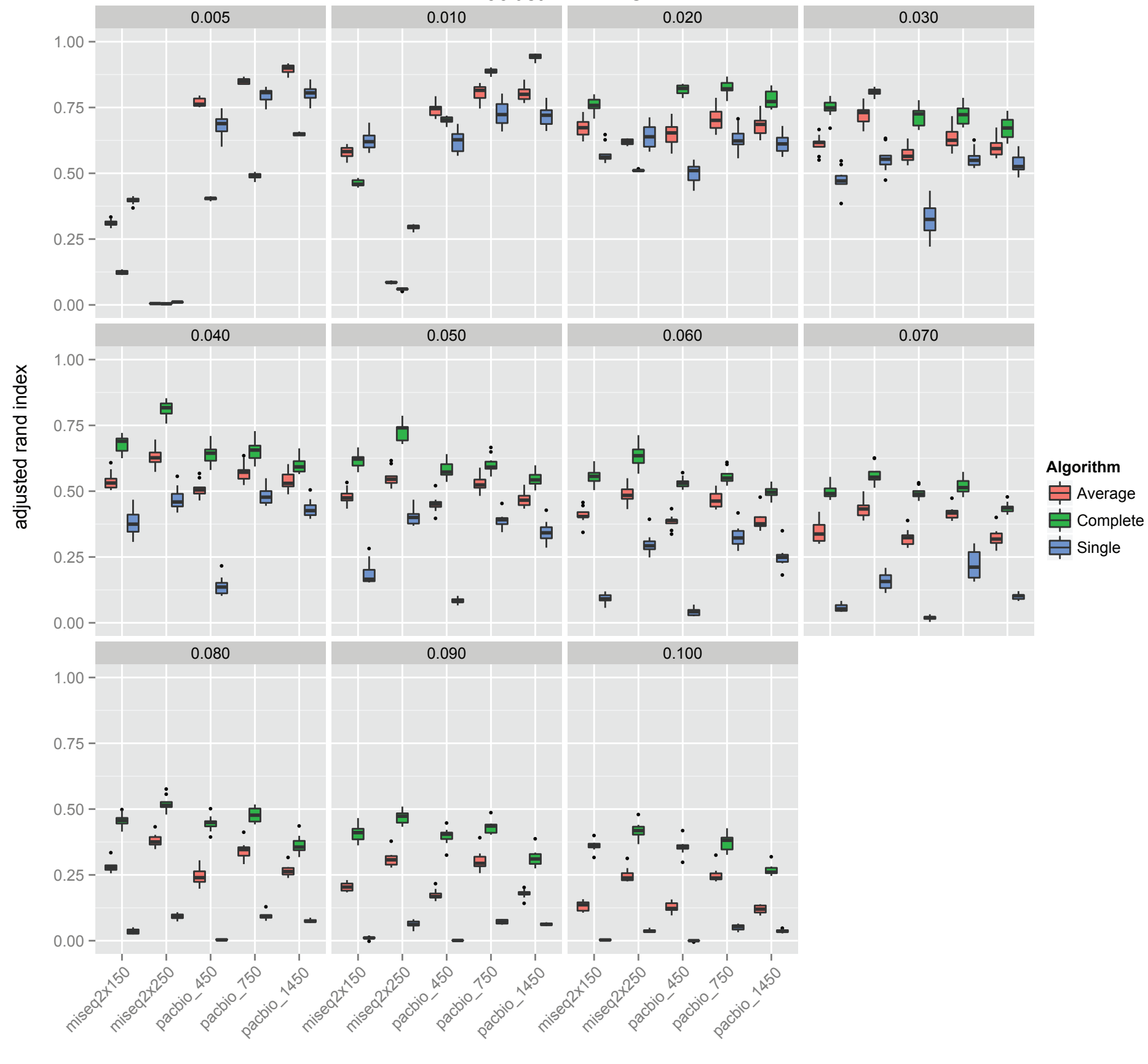

# oclust PW - HC

adjusted rand index

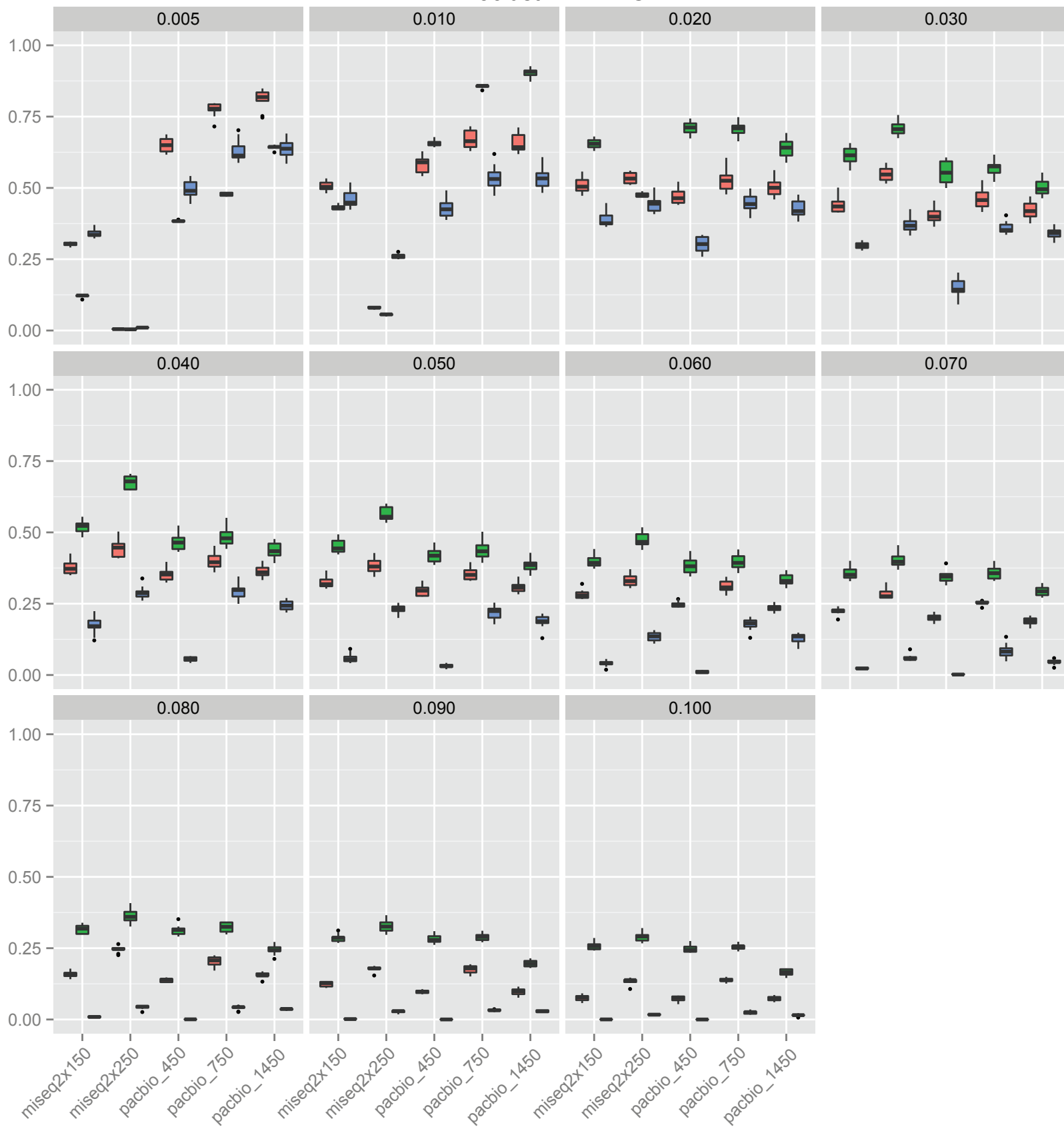

Supplement: Additional file 1: — Comparison of hierarchical clustering algorithms. Average- (AL), complete- (CL), and single (SL)-linkage hierarchical clustering were evaluated on low, medium, and high complexity mock communities using the adjusted Rand index (ARI; y-axis). The clustering outcome was then evaluated at 11 distance thresholds (0.5–10 %). Red, green, and blue colors correspond to AL, CL, and SL, respectively. [file 40168_2015_105_MOESM1_ESM.pdf]

evaluated clustering program: dnaclust

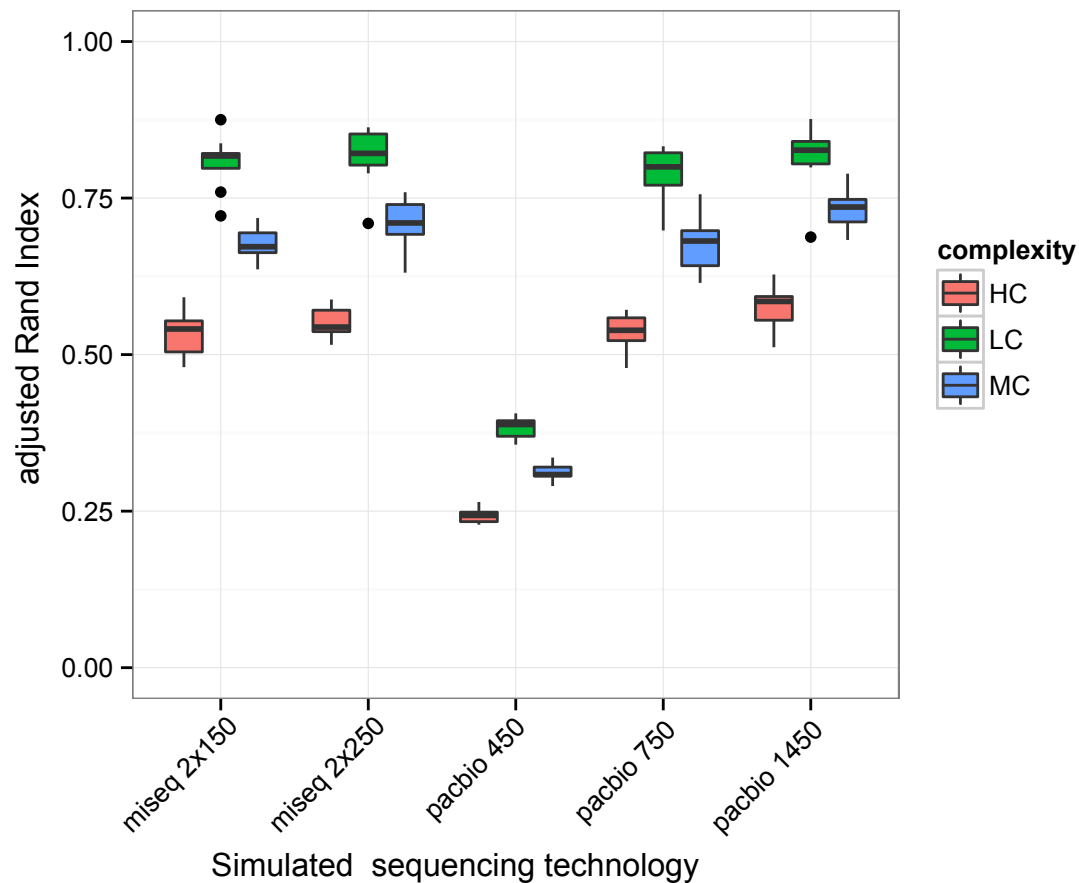

Supplement: Additional file 3: — Clustering accuracy of simulated sequencing on mock communities using DNACLUST. Clustering accuracy was measured with the adjusted Rand index score (ARI; y-axis) on five simulated sequencing read lengths by DNACLUST. The performance of DNACLUST is similar to the two other heuristics at all complexity levels. [file 40168_2015_105_MOESM3_ESM.pdf]

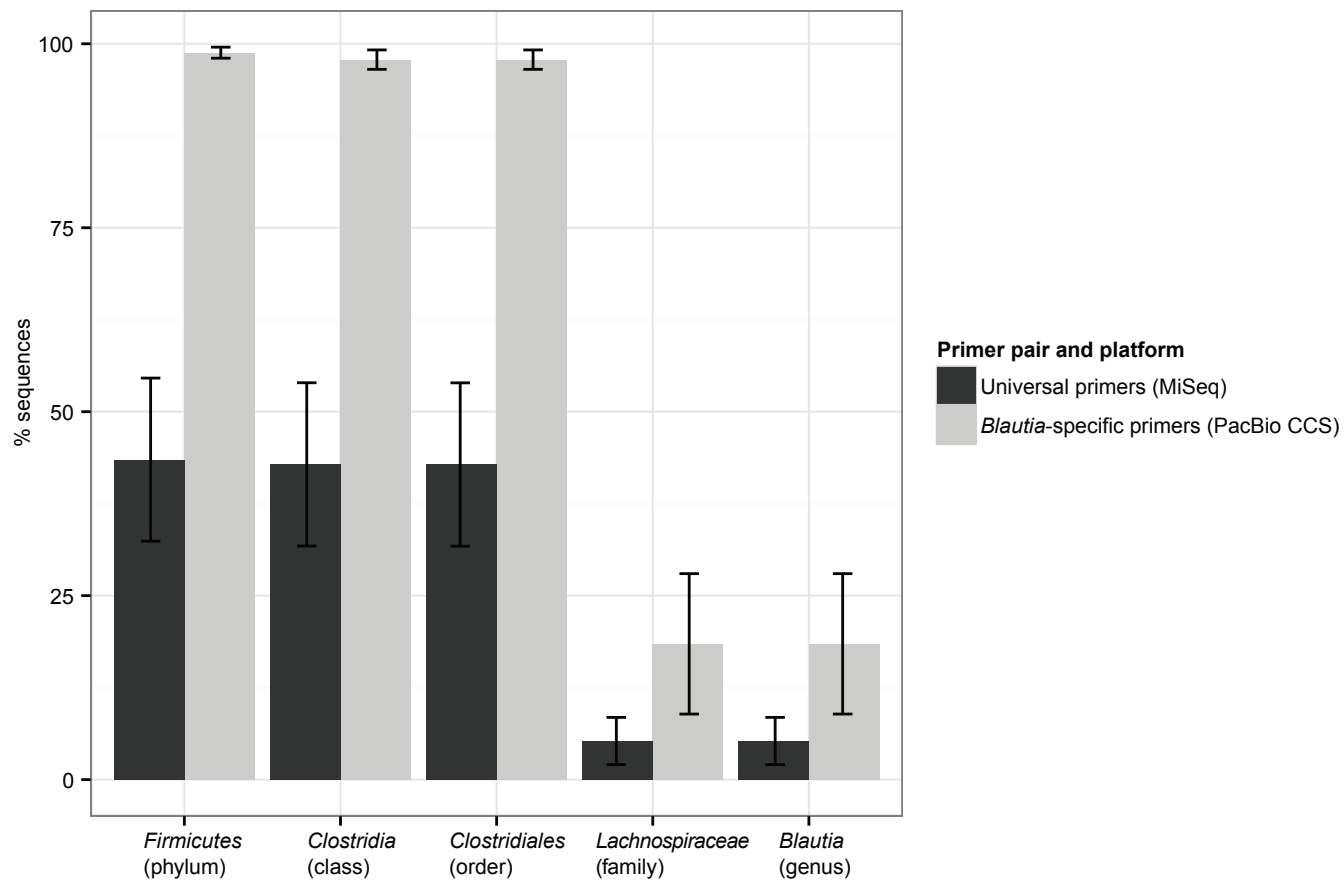

Supplement: Additional file 4: — Taxonomic enrichment achieved within Blautia. The y-axis shows the mean percentage reads classified to a specific taxonomic rank per sample. Error bars correspond to the standard deviation across samples. The x-axis shows the taxonomic rank along the Blautia lineage. Taxonomic classification was performed with QIIME (scores >0.8 were considered correct). Black and gray colors refer to universal primers and Blautia-specific primers, respectively. [file 40168_2015_105_MOESM4_ESM.pdf]

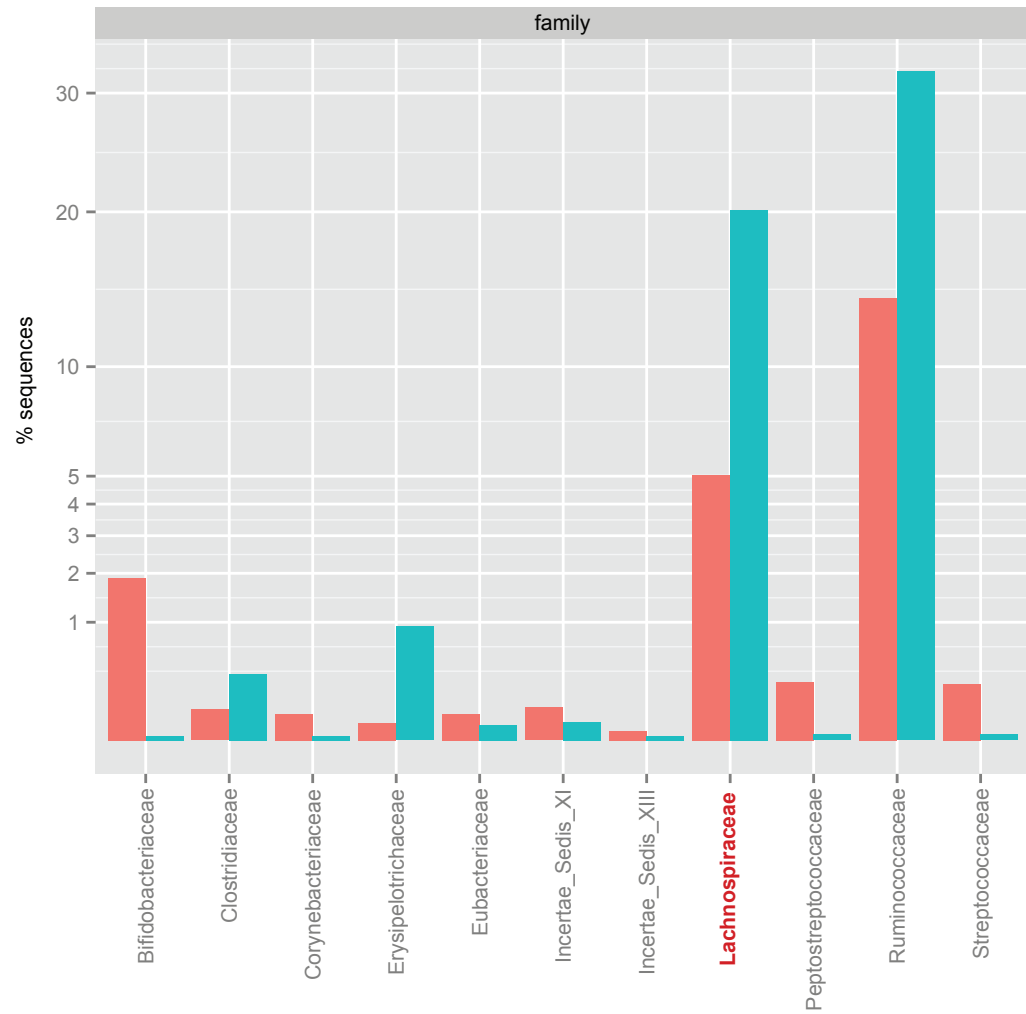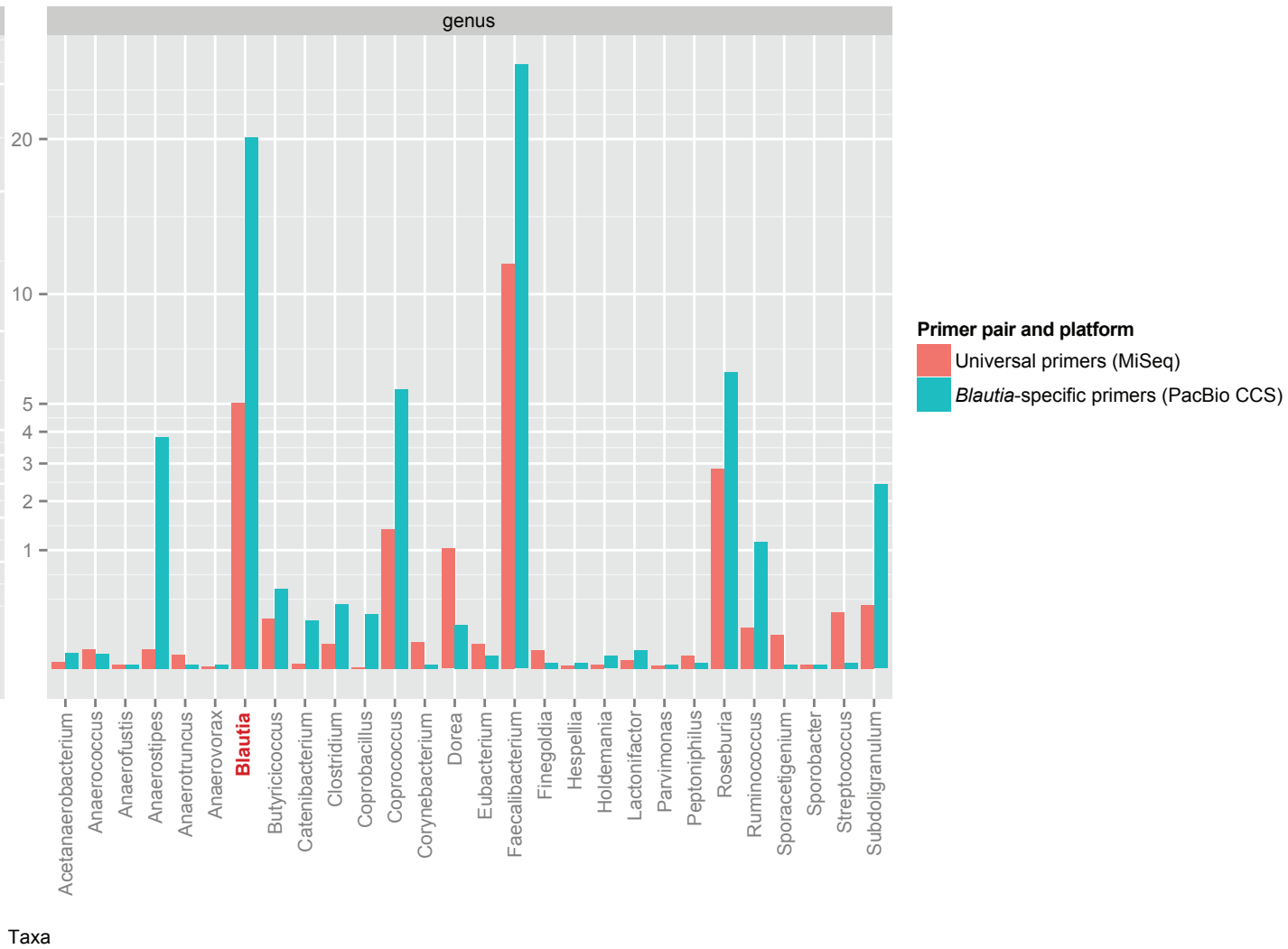

Supplement: Additional file 5: — Non-specific taxonomic enrichment. Bar plots showing enriched taxa for sequencing using universal 16S rRNA gene primers (MiSeq) as compared with Blautia (genus)-specific primers (PacBio CCS). The y-axis refers to percentage of sequences in all samples. The x-axis refers to the specific taxa. The left plot shows taxa at the family level, and the right plot shows taxa at the genus level. Only taxa with abundance >0 using universal- and Blautia-specific primers are shown. Data (y-axis) were square root transformed prior to plotting. [file 40168_2015_105_MOESM5_ESM.pdf]

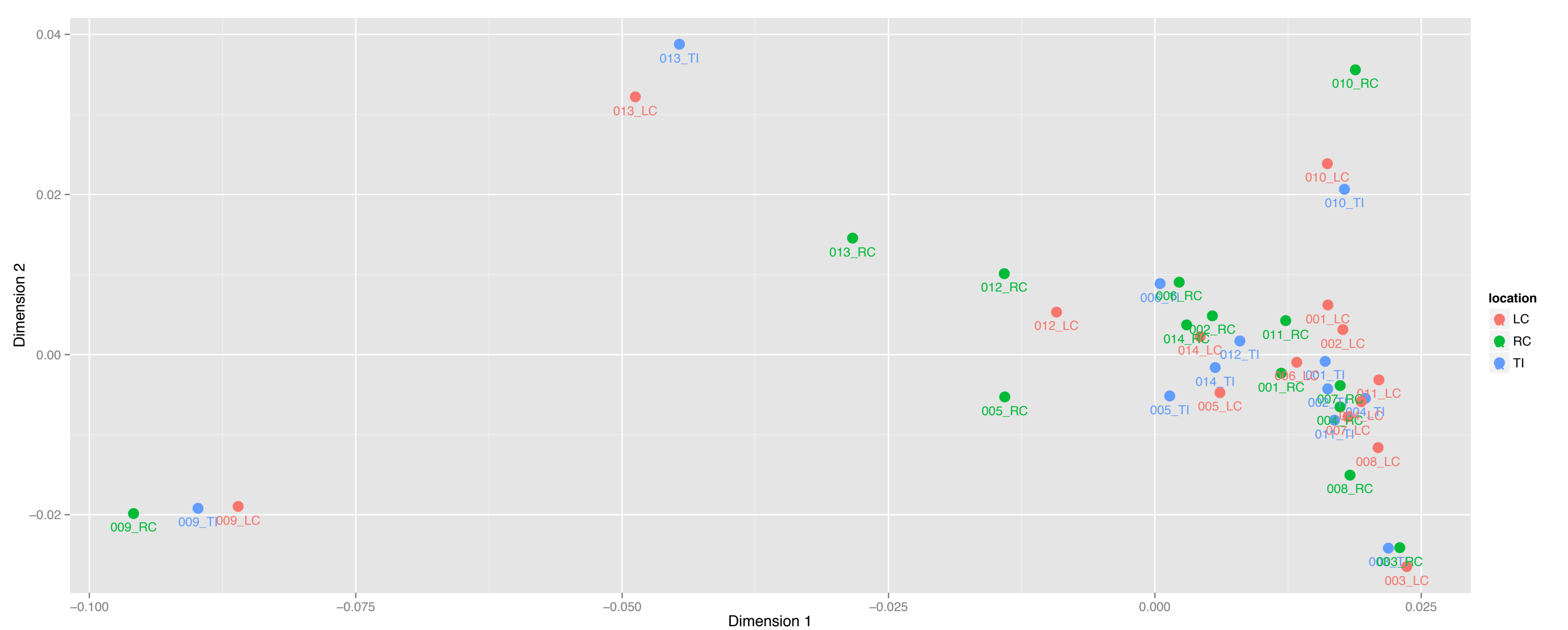

Supplement: Additional file 7: — Multidimensional scaling plot of intestinal sample locations versus OTU profiles. Blautia OTU profiles were computed with oclust PW (sequence similarity = 3 %), and multidimensional scaling was performed in R using the cmdscale command. Colors refer to the intestinal location (red = LC, green = RC, blue = TI). Sample identifiers are plotted below each point. [file 40168_2015_105_MOESM7_ESM.pdf]

0.01

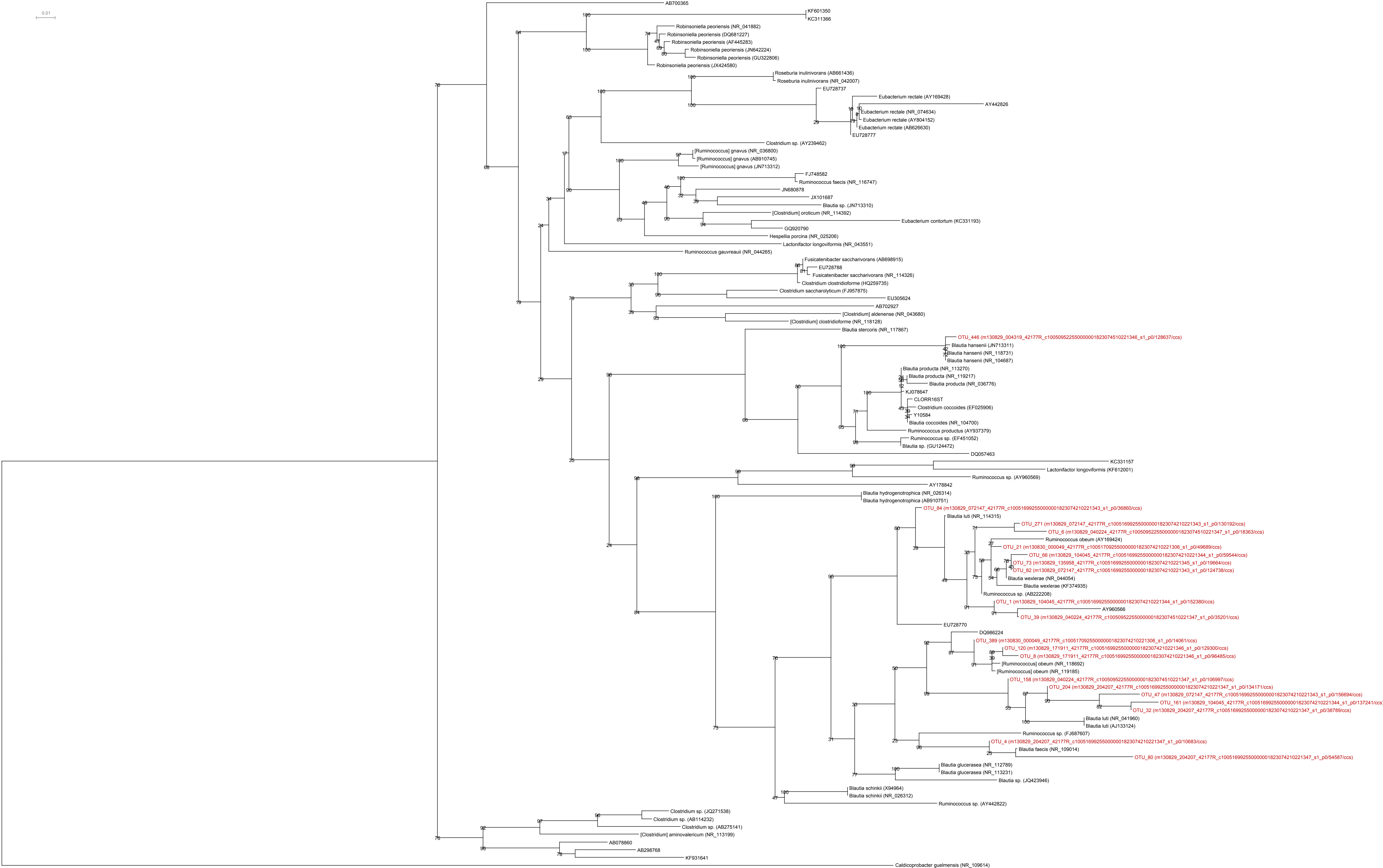

Supplement: Additional file 8: — Maximum likelihood phylogenetic tree of representative 16S rRNA gene sequences of Blautia OTUs. One representative sequence from each OTU was selected using the minimum genetic distance. Closely related full-length 16S rRNA sequences were identified from NCBI GenBank. A multiple sequence alignment was created with clustalw2, and the phylogeny was inferred with RAxML with the GTRGAMMA nucleotide substitution model. Node labels indicate bootstrap support values. If the reference sequence from GenBank had meaningful taxonomic information (e.g., binomial species name), it is shown on tips; otherwise, only the accession number is shown. Square brackets around a genus name indicate a candidate genus. The top left scale bar indicates number of substitutions per site. The tree was rooted using Caldicoprobacter guelmensis (NR_109614). Blautia OTUs are indicated in red and the identifier of the representative PacBio CCS read is shown in parenthesis. [file 40168_2015_105_MOESM8_ESM.pdf]
